# An Anthropological Analysis of Acceptability and Feasibility of Expanding Community-Based Malaria Management to All Ages in Madagascar: Levers and Challenges for National Scale-Up

**DOI:** 10.4269/ajtmh.24-0678

**Published:** 2025-08-12

**Authors:** Hoby Fenitra Rabesandratra, Chiarella Mattern, Emilia Brazy-Nancy, Aina Harimanana, Judickaelle Irinantenaina, Hobisoa Léa Razanadranaivo, Pierrette Tianiaina Daniella Andrianambinintsoa, Catherine Dentinger, Laura Steinhardt, Andres Garchitorena

**Affiliations:** ^1^ Santé et Sciences Sociales, Institut Pasteur de Madagascar, Antananarivo, Madagascar;; ^2^ Unité d’épidémiologie et de recherche clinique, Institut Pasteur de Madagascar, Antananarivo, Madagascar;; ^3^ Malaria Branch, National Center for Emerging and Zoonotic Infectious Diseases, Centers for Disease Control and Prevention, Atlanta, Georgia;; ^4^ UMR MIVEGEC, IRD, CNRS, Université de Montpellier, Montpellier, France

## Abstract

Despite significant progress in reducing malaria effects in recent decades, malaria remains a major challenge in Madagascar. Geographic and financial barriers often prevent individuals from seeking prompt care. Community health workers (CHWs) in many countries, including Madagascar, provide malaria case management services to children younger than 5 years old, although they typically do not treat older children and adults, leaving a gap for those living far from health facilities. To determine the efficacy of expanding malaria community case management (mCCM) to community members of all ages, a cluster randomized trial was conducted in one district of Madagascar from November 2020 to December 2021. Qualitative surveys were conducted to describe the acceptability and feasibility of this intervention among beneficiaries and CHWs. For this purpose, 87 semistructured interviews and 12 focus groups were conducted in intervention and control arms of the study to assess understanding of malaria, behaviors related to care seeking for fever, perceptions of CHW roles, and acceptability and feasibility of the age-expanded mCCM. Two major findings emerged. First, stakeholders found age-expanded mCCM to be consistent with existing CHW roles and practices. Age-expanded mCCM induced a recognition of adults’ susceptibility to malaria and led to a more accurate understanding of malaria. Second, structural and community-based challenges were not fully resolved by age-expanded mCCM, and some, such as the question of the cost of care, emerged after its implementation. Despite the fact that age-expanded mCCM was acceptable to beneficiaries and CHWs, successful scale-up will require addressing structural challenges and sociodemographic inequalities.

## INTRODUCTION

Despite significant progress in reducing malaria morbidity and mortality since 2000, some endemic regions are facing challenges to sustain this progress, and efforts have stalled or reversed in recent years, particularly in Africa.^1^^,^^2^ Improving universal access to malaria diagnosis and treatment, one of the pillars of the WHO’s Global Technical Strategy, could help address this,^2^ but barriers to accessing care have been particularly persistent in rural endemic areas.^3^ To reduce geographic and financial barriers, several countries have implemented integrated community case management (iCCM) programs, whereby community health workers (CHWs) diagnose and treat common illnesses, including malaria, among children younger than 5 years of age (CU5). Although iCCM has increased access to care and reduced child mortality,^4^ it has rarely been expanded to include older age groups, leaving most remote populations with poor access to malaria care.

Madagascar is illustrative of the challenges facing many endemic countries in sub-Saharan Africa, and national malaria cases have more than doubled between 2019 and 2023.^5^ The national strategy to combat malaria relies on a combination of disease prevention activities, such as distribution of long-lasting insecticide-treated bed nets and indoor residual spraying of insecticides in limited areas, together with free access to malaria diagnostics and treatment. Under the national iCCM program, CU5 presenting with fever can be diagnosed by CHWs with rapid diagnostic tests (RDTs) and treated with an artemisinin-based combination therapy (ACT) if malaria is confirmed. Despite these efforts, the malaria burden remains high in Madagascar, with more than 2 million cases reported per year.^6^ Moreover, recent studies have shown that individuals older than 5 years old, especially those ages 5–14 years old, have the highest prevalence of parasitemia.^5^^–^^7^ As a result, the National Malaria Control Program included the expansion of malaria community case management (mCCM) to all ages (age-expanded mCCM) in its 2018–2022 strategic plan. Given the lack of robust evidence for age-expanded mCCM in Madagascar or elsewhere, a cluster randomized, controlled trial (RCT) was conducted in one district in southeastern Madagascar before nationwide implementation^7^^,^^8^ to understand the impact of this intervention on care seeking and malaria prevalence.

To obtain a context-specific understanding of the implementation process and assess the acceptability and feasibility of the intervention to community members (beneficiaries), CHWs, and facility-based health care workers (HWs), a qualitative component was included in the age-expanded mCCM study.^8^^,^^9^ Indeed, understanding the social factors that influence the course, acceptability, and impact of an intervention is an essential step before scaling up to regional or national levels.^9^^,^^10^ Implementation research embedded within randomized trials allows for an in-depth understanding of social phenomena and mechanisms through descriptions of care provision and practices, and it sheds light on the social norms governing access to care and whether interventions are assimilated by the population.^11^ Furthermore, comparing results from populations in the intervention and control arms of the trial provides contextualized information on the intervention impact.

## MATERIALS AND METHODS

### Background on the mCCM trial.

This qualitative research was conducted as part of a cluster randomized trial (PACTR202001907367187)^8^ to assess the effect of age-expanded mCCM on care seeking and malaria case management. The trial was conducted in Farafangana, a rural health district located in southeastern Madagascar with moderate malaria transmission according to routine surveillance data (79.2 cases per 1,000 persons in 2017). The population of Farafangana consists of nearly 500,000 people served by 38 public health care centers (PHCs), whose staff are responsible for supervising more than 600 CHWs. Geographic accessibility to primary care in the district is poor, with less than 40% of the population living within 5 km of a PHC.^12^ The trial encompassed the catchment area of 30 PHCs and their corresponding 502 CHWs.

For the trial, PHCs and their corresponding CHWs and catchment areas were randomized 1:1 to either the intervention arm, where CHWs implemented age-expanded mCCM (intervention) that provided malaria testing and treatment to community members of all ages in addition to iCCM for CU5, or the control arm, where CHWs conducted iCCM for CU5 according to national policy. Before the intervention, all CHWs received a refresher training on iCCM, and those from the intervention arm received additional training on age-expanded mCCM. A community sensitization campaign conducted in October 2020 included meetings with local authorities and local radio broadcasts to inform the population of the intervention and remind them that mCCM was free of charge. After the intervention was launched and it became apparent that some CHWs might be charging a small fee for malaria commodities to compensate for their labor and lost income, trial funds were used to provide each CHW with a stipend of 15 U.S. dollars every 6 months during the study period. In addition, study staff strengthened CHW supervision, including for completing disease reporting and stock management tools. To assure adequate supplies during the trial, the malaria commodity supply chain was heavily reinforced in both intervention and control arms, including quantification of malaria commodities, storage of additional commodities in the district, and transport from PHCs to communities. Implementation of age-expanded mCCM occurred from November 1, 2020 to December 31, 2021 for a total duration of 14 months.

### Study objectives and design.

One of the main objectives of the mCCM trial was to understand the acceptability and feasibility of expanding community-based malaria management to individuals older than the age of 5 years old, including the factors and implementation challenges that need to be considered for nationwide scale-up of this initiative. For this, a qualitative study was conducted in the two study arms through semistructured interviews among beneficiaries, CHWs, and HWs. In addition, focus group discussions among beneficiaries and CHWs were conducted in the intervention arm. Besides this analysis of acceptability and feasibility, a secondary goal was to describe the impact of the intervention on care seeking for fever, factors influencing health care choices, illness representations, and interactions between beneficiaries and health care staff by comparing results from populations in the intervention and control arms. Themes that emerged from study data were explored through successive rounds of discussion between implementation teams and researchers.^13^

This anthropological analysis of malaria care aims to contribute to the broader field of implementation science, which examines methods and strategies for effectively implementing evidence-based interventions, practices, or policies in real-world settings. Two thirds of clinical trials do not succeed because they are not embraced by communities,^14^ often because of insufficient analysis of the social aspects of the interventions or the context in which they are carried out.^15^ The primary goal of implementation science is to understand how scientific knowledge can be transformed into tangible actions to enhance health, social services, and other sectors,^14^^,^^16^ and anthropology is particularly well placed to address intricate questions regarding how and why attempts to implement best practices may either succeed or fail as well as to identify the social dynamics that influence these outcomes.^17^ The combination of methods used here (focus groups and semistructured interviews) is considered as an effective approach for gaining insights into beneficiaries’ perceptions of the innovation and the implementation efforts.^17^

**Figure 1.**
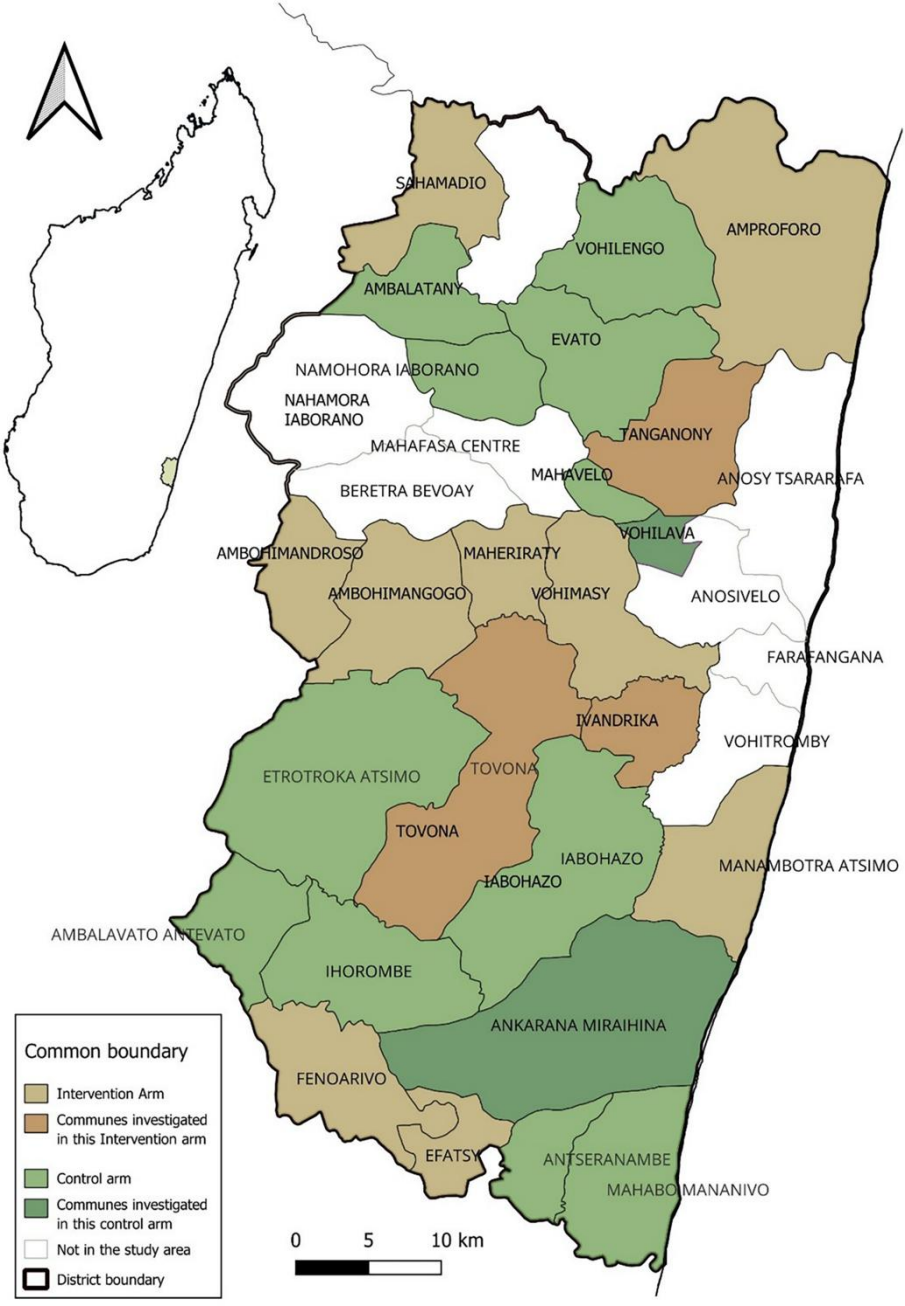
Map of the malaria community case management cluster randomized trial study area with the communes included in the qualitative study. Intervention and control arms are represented in brown and green, respectively. Communes included in the qualitative study are represented with darker colors, and those not included are represented with lighter colors.

The study took place in five communes: three in the intervention arm and two in the control arm (Figure 1). These communes were selected based on whether the trial coordinators and their implementation team had observed organizational or relational challenges in malaria case management and/or implementation of the intervention (such as pre-existing tensions between CHWs and HWs or a proven difficulty in the management of supplies by CHWs) based on the inputs of the trial’s field supervisors during four separate interviews. These included hypotheses such as potential adult care taking place in the control group, a high perceived effectiveness of RDTs and ACTs by community members, and some challenges identified by the team in the intervention group.

Within each commune identified, one fokontany (the smallest administrative division in Madagascar comprising one or several villages) was selected among those located more than 5 km from the closest PHC, excluding those that presented security concerns or where the fokontany chief refused participation. A security incident during fieldwork in one commune of the intervention arm led to suspension of some activities there and replacement with a commune of equivalent characteristics to continue the data collection. Thus, three communes were included in the intervention arm instead of two.

### Data collection.

Data were collected through semistructured interviews and focus group discussions among stakeholders from October 16 to December 23, 2021 by a team consisting of two anthropologists, a research assistant, two interpreters, and a French–Malagasy translator. To enable comparison, similar themes were addressed in both study arms; however, in the control arm, greater emphasis was placed on health system limitations in the management of malaria, and in the intervention arm, feedback on the intervention was emphasized. Beneficiaries’ opinions about age-expanded mCCM and its impact on their practices and daily lives, even among those in the control arm, were elicited. Other themes addressed related to malaria case management practices, care seeking for fever, beneficiaries’ understanding of the intervention, relationships between stakeholders (HWs, CHWs, and beneficiaries), and organization of the health care system.

Participants for interviews and focus groups were recruited by convenience based on their age, the age of their children, and their availability. This was done to assure inclusion of adults ages 20–35, 36–50, and older than 50 years old whose family composition included both those with young children (CU5) and those with older children. To limit anchoring bias,^18^^–^^20^ beneficiaries were approached by administrative officials (fokontany chiefs) and not CHWs.

Methodological triangulation^21^ was performed by combining data from the semistructured interviews and the focus groups. Data collection proceeded iteratively using feedback from the data obtained to adjust the interview guides.^18^ Interviews and focus groups were conducted in Malagasy and recorded for quality control and translation purposes. Discussions were led by a facilitator and an observer who noted the details of the focus groups and could suggest follow-up questions.

### Data analysis.

The semistructured interviews and focus group recordings were transcribed, translated into French, and subjected to a thematic analysis using grids designed for each category of participant to highlight recurrences and divergences in the discussions. These recurrences and divergences were then analyzed to assess the acceptability of the intervention and to describe the practices pertaining to malaria in terms of health care use and the changes that the participants perceived and attributed to the intervention.

## RESULTS

In total, 87 semistructured interviews and 12 focus groups were conducted (Table 1). Two major findings emerged from the data analysis. One concerned the acceptability and feasibility of the mCCM intervention among stakeholders, and the second concerned the challenges, both structural and community based, that were not resolved by the intervention or that emerged after its implementation (Supplemental Appendices 1–8).

**Table 1:** Summary of interviews and focus groups conducted according to study arm and population group

Semistructured Interviews among Stakeholders	Control Arm	Intervention Arm
Beneficiaries* (community members)	20	24
CHWs	11	12
Health care workers	10	10
Total	41	46

Focus groups		
Beneficiaries (community members)	0	8^†^ (total of 48 community members ages 20–35, 36–45, and older than 45 years old in separate groups)
CHWs	0	4 (total of 24 CHWs, all ages)
Total	0	12 focus groups (60 persons)

CHW = community health worker.

* The selection of participants in the community member group was designed to ensure parity between male and female genders.

^†^ There were six individuals in each focus group. There were three focus groups for persons between ages 20 and 35 years old, three focus groups for persons between ages 36 and 45 years old, and two focus groups for persons older than 45 years old.

### A mostly well-accepted strategy by stakeholders.

Most stakeholders considered the expansion of community-based malaria management to people older than 5 years old as acceptable based on three findings. 1) It conferred direct positive effects to beneficiaries, 2) it appeared to be consistent with usual care-seeking practices and malaria case management, and 3) it resolved certain challenges encountered in current (usual) malaria management practices. However, a few HWs, mainly those in the control arm, expressed doubts about the skills of certain CHWs to manage supplies (e.g., RDTs and ACTs) appropriately and without diversions and to identify contraindications to ACT use in adults. In addition, some HWs expressed concerns that CHWs might feel empowered to provide care for diseases other than malaria, which would be inappropriate. CHWs in the intervention arm declared that they were generally satisfied with the intervention and would like to continue managing malaria in older children and adults. As for the CHWs in the control arm, they expressed the wish to be allowed to do the same.

#### A strategy with direct positive effects.

Several direct positive effects of age-expanded mCCM were identified in the intervention arm. First, there were reduced tensions between CHWs and beneficiaries as the ability to consult with CHWs was widely appreciated by adults. They were grateful to be able to take children older than 5 years old and teenagers to CHWs and to be allowed to go themselves. Indeed, tensions and misunderstandings emerged between community members and CHWs when only CU5 were accepted for care. Mothers and CHWs frequently reported situations where a mother criticized the CHW for admitting one child while refusing her own, despite the fact that both children were approximately the same size. There were also instances where a child who had previously received care from a CHW was turned away at the age of 6 or 7 years old. As one CHW expressed, “It’s challenging, especially when a mother brings in her sick child and asks wryly, ‘What’s the difference between his body now and before? Can’t he die of malaria anymore as soon as he’s 6?’” (CHW, female, 35 years of age, control arm). CHWs described themselves being caught between obligations to their communities and their own moral standards. Because of the intervention setup, the CHWs in the intervention arm no longer felt torn between their commitment to respecting Ministry of Public Health policies and their fears that refusing care could result in more severe illness or death of older children and adults who had come to them for help, which could lead to tensions or even reprisals against CHWs from community members.

Before, when we [CHWs] didn’t treat them [beneficiaries older than 5 years old], they said we discriminated against people, yes … it’s because we’re haughty so, for them, we don’t like them in fact and that’s why we don’t treat them. When that finally happened [the mCCM intervention] then it was … ahahaha (laughs), they were finally motivated, as soon as they got the temperature up, they’d come straight away, “let’s go to there to the CHWs,” they’d say, right away they saw us differently, that’s how it is for them. (CHW, farmer, female, 27 years of age, intervention arm)

Stakeholders also recognized a strengthening of ties between HWs and CHWs because of the frequent interactions and increased supervision in both arms. HWs at PHCs noted a reduction in their workload. They now felt able to manage other diseases rather than just running a “dispensary dedicated to malaria” as noted by one HW in the intervention arm.

A second positive effect of age-expanded mCCM was a notable improvement in malaria knowledge and prevention practices among beneficiaries in both arms. The mCCM intervention resulted in a better understanding of adults’ risk of malaria and the link between malaria and its mosquito vector, and it resulted in less attribution of the disease to magical or religious sources.

Narratives tracing the history of malaria in the region show that the introduction of iCCM in Farafangana triggered an understanding of the association between malaria as a disease and the symptoms formerly called mamo^†^ or fanintona.^‡^ Before iCCM and related community sensitization were introduced, the term “malaria” was not generally known or used, and malaria was not associated with mosquito bites. Rather, it was associated with witchcraft or a curse. Although the understanding of the cause of malaria improved, it was widely considered to be a disease mainly affecting CU5, and malaria symptoms in adults tended to be attributed to fatigue, malnutrition, or resulting from witchcraft.

According to study interviews and discussions, age-expanded mCCM led a large portion of the population to realize that malaria could also affect teenagers and adults as RDT results made it visible to them. This realization was observed in both study arms. The population also observed that taking an ACT (after a positive RDT) improved symptoms.

According to CHWs and HWs, before the mCCM intervention was implemented, the majority of people older than 5 years of age with fever were treated with medicinal plants and/or traditional medicines given by ombiasy (traditional healers) before seeking care from a health facility. Some members of the community as well as certain health personnel revealed to us that certain people older than the age of 5 years old were cared for by the CHWs on an ad hoc basis, disregarding the national recommendation.

Study participants indicated that care-seeking delays were reduced with age-expanded mCCM from several days to less than 24 hours, thus reducing therapeutic meandering (a situation where a patient self-medicates and/or seeks care at multiple providers before consulting the health system, often because of various obstacles that make their care journey complex or even chaotic),^22^^,^^23^ which can lead to worsening disease or death. In the event of symptoms associated with malaria (e.g., fever, rigors, etc.), CHWs and HWs reported that patients requested an RDT to check for malaria, which they were able to do now in the community. This improved knowledge of the disease appeared to have had an impact on practices.

[Before mCCM,] they told themselves they would be better in the morning, and they make do with traditional Malagasy methods such as infusions of the leaves of certain plants, which they boil. If this doesn’t prove effective, their case can worsen and they end up dying. (CHW, farmer, male, 32 years old, control arm)

And the people who used to go to the ombiasy [traditional healer], what have they been doing since the intervention? They still go, except for malaria. When they’re sick, they come to us to be tested with the RDT, and if it’s positive they take the ACT, but if it’s negative, they go back to the ombiasy. (CHW, farmer, female, 27 years of age, intervention arm)

Before it was not in the first 24 hours but really only after 2 or 3 days that people came to do a test but very few people do that now. (midwife in a PHC, 37 years of age, intervention arm)

Our analyses also showed a tendency for some adults and older children to seek care from CHWs long before the trial existed. This suggests that some community members understood that adults could become infected with malaria and attributed the possibility of recovery to a CHW’s care. For these participants, age-extended mCCM helped to confirm what they suspected.

#### A strategy consistent with usual practices.

Age-expanded mCCM was perceived to be an extension of the well-established and accepted practice of iCCM for CU5 and thus, was well accepted in the intervention arm. Furthermore, control arm participants expressed frustration that they were not also receiving the intervention, suggesting that it would be acceptable there. Control arm CHWs were accepted as the entry point to the health care system in the event of fever among CU5, whereas older individuals experiencing fever practiced home management using drugs purchased at the local market and occasionally consulted with CHWs, depending on their relationship with the CHW. All categories of stakeholders in the control arm acknowledged a tendency of therapeutic meandering among older children and adults, which could last several days, and noted that this practice could delay care seeking at PHCs. Care-seeking delays and therapeutic meandering were attributed to poverty, distance from PHCs, and difficulties differentiating malaria from “simple” fever as well as PHC consultations not being free of charge.

Some respondents reported that there had been unofficial care seeking from CHWs for fever and suspected malaria among older children and adults before the trial. For example, they noted that children ages 6–15 years old were frequently taken to CHWs for fever, and rather than refer these older children to a PHC per the national policy, the CHW would test them for malaria and treat them if positive. CHWs might be willing to go beyond their scope of practice to care for this age group of children. Adults, on the other hand, tended to seek care from traditional practitioners or to buy drugs, such as amoxicillin, in the local market.

Several reasons were given for preferring CHWs to manage malaria for patients of all ages. CHWs were perceived as more accessible (i.e., lived close by, available around the clock). The logistics of CHW services were preferable (e.g., home visits if necessary; various payment options, including credit, installments, or in kind). The quality of the relationship with CHWs was generally perceived as high, and CHWs afforded better social proximity. (Social proximity refers to patterns of friendship, partnership, and cultural behaviors^24^ resulting from frequent interaction and similar ethnic, linguistic, and experiential origins and levels of education.) Economic insecurity, fear of hospitalization, a general distrust of the formal health system, and additional services offered by CHWs because of neighborly relationships, such as keeping the child for observation, also favored a preference for CHWs.

Our CHW accepted my child late in the evening, and given the insecurity, she did not let us go home alone with a sick child. We stayed at her place; she served us sweet tea and fed my child. We left the next day. (community member, farmer, female, 30 years of age, intervention arm)

Moreover, there are cultural and socioeconomic gaps between community members and HWs, largely because of differences in their background and education, as they are typically assigned to these areas and hold specific qualifications. CHWs thus serve as a crucial bridge between health centers and the population, being regarded as “children” and “allies” of the health worker at the PHC as well as respected and listened-to figures within the community.

They consider me a “vazaha” (of foreign nationality) because of the difference in lifestyle, education I suppose. They [members of the community] say out loud: “She’s too clean, she can’t stand living here among us.” (doctor at the PHC, female, 42 years of age, control arm)

CHWs have seen their image enhanced among the population because of the trust that was placed in them during the mCCM intervention. CHWs were felt to be the right people in the right place when it comes to managing malaria in their locality. Most beneficiaries and HWs felt that CHWs were capable of managing uncomplicated malaria cases; however, the inconsistent availability of essential supplies to test for and treat uncomplicated malaria was cited as a barrier to getting care from them.

In short, age-extended mCCM was perceived as a formalization of usual care-seeking practices in these rural communities as opposed to a new service, and as such, it was widely accepted. The management of malaria by CHWs, despite their lack of formal academic training in medicine, proved to be a highly acceptable practice, was embraced, and was even preferred by community members.

#### A strategy offering solutions to existing challenges in mCCM.

The introduction of the mCCM has resulted in advancements in two of the most commonly mentioned issues in malaria management in Madagascar: self-medication practices^25^^,^^26^ and supply chain challenge^4^^,^^27^ in areas comparable with our study region.

Self-medication practices characterize the relationship between community members and malaria. This results, among other factors, from a cultural aspect, notably the nonexistence of malaria as a clinical disease but rather, attributed to fatigue, especially in adults, or the geographical distance from health centers, prompting patients to seek alternative solutions. Interviewed participants noted their intention to practice less self-medication for three main reasons directly linked to the mCCM intervention. First, because of the strengthening of the supply chain, ACTs could now be obtained from CHWs and PHCs in both intervention and control arms. Second, community members became aware of the harm of self-medication and the importance of seeking care in the formal health system (PHCs and CHWs) in part because of sensitization messages delivered during the intervention. Third, the perceived efficacy of malaria management by CHWs and HWs was attributed to the use of RDTs to confirm their infection and to the quality of the medicines offered by these providers as opposed to products purchased at the market. Some interviewees noted a rapid improvement after ACT treatment, which encouraged them to seek care with CHWs and HWs rather than practice self-medication; they associated improvement with the ACTs acquired from CHWs and PHCs.

The medicines they [CHWs and HWs] give are effective. No one treated by the doctor (here, a CHW), who received medicine for malaria from the doctor, has ever died. This has not [yet] happened. We are really in good health once we get the medication. (37-year-old community member, male, during a focus group of men ages 35–45 years old, intervention arm)

The second major challenge identified was related to the supply chain, specifically the lack of RDTs and ACTs at the community level. During the trial, the malaria supply chain was strengthened in both control and intervention arms, and all participants identified this as a benefit of age-expanded mCCM. Stakeholders noted that the time between symptom onset and treatment decreased because CHWs had the supplies that they needed to provide care rather than having to refer beneficiaries to a PHC. The greater availability of these supplies was a major determinant in beneficiaries’ choice to consult CHWs.

Since the trial set up by the Institut Pasteur, ACT has been continually available because there is a monthly distribution of “ACT” and “RDTs.” There have been no stock outs in the supply since March. (CHW and farmer, male, 35 years of age, control arm)

Stakeholders generally agreed that improved availability of malaria commodities was a benefit of age-expanded mCCM; however, some noted that there were still periodic mCCM supply shortages.

### Persistent and novel challenges observed in the intervention arm.

Some challenges related to mCCM were present before the age-expanded mCCM trial and persisted despite it. New challenges also emerged after implementation.

#### Persistent frustration with theoretically free access to malaria care.

First, care for malaria was not always free. Patients in the two arms were charged a variable fee for their care, and many interviewees and focus group participants in the intervention arm reported a cost increase once expanded-age mCCM began. A CHW might charge different prices for different families during the same period. These fees were charged for two main reasons. First, patients often misunderstood the difference between supplies (e.g., RDTs and ACTs), which were free, and the visit, which might include vitamins, antipyretics, and other services provided by CHWs and PHCs. Having heard in the media that the malaria commodities were free in both study arms, beneficiaries were not expecting to be charged for CHW treatment. Second, when malaria commodities were occasionally unavailable in part because of the larger number of patients consulting and supply chain failures, CHWs purchased supplies from local depots and pharmacies and passed the costs to patients. This could explain why members of the same family might be charged different prices. HWs also reported rare cases of CHWs misappropriating materials, including for resale in private markets.

The CHW-level costs appeared to concern mainly the care of the adults, not older children. During focus group sessions, participants compared the prices that CHWs had charged them and realized that different prices were being charged. The CHWs appeared to set their prices according to the economic status of the patients. Interviewees reported that children and adults with a modest income paid less than cattle owners and families deemed to be more affluent. This led to frustration about the lack of standardized costs and price transparency at the community level.

#### Unintended impacts of mCCM expansion on CHWs and HWs.

All stakeholders noted that age-expanded mCCM increased the CHW workload, resulting in less time for CHWs to pursue income-generating activities. The increased workload was because of more patients but also, because of the increase and complexity of reporting requirements. Many CHWs complained about their working conditions, particularly that they were unpaid.

What has made our workload heavier is that we are called on when we are engaged in our subsistence activity. We postpone work until the afternoon. Work is interrupted. (CHW, farmer, female, 45 years of age, intervention arm)

The additional CHW workload had a negative impact on their availability for engagement in other programs, such as sensitization and awareness campaigns. Health personnel, for example, noted a neglect of vaccination awareness activities and efforts to reach zero-dose (unvaccinated) children, which is an important part of their work.

What I have observed is that vaccines are rather neglected, they are too distracted. Before, if there were unvaccinated children, we told them [CHWs] to go get them and they would bring them back to the next meeting, but that is no longer the case. I think their schedule must be busier, and that has consequences for vaccines. (nurse at PHC, female, 28 years old, intervention arm)

Health workers at PHCs reported increased costs incurred because of the intervention, particularly for transporting additional supplies for CHWs in their catchment areas from the district pharmacy in Farafangana to the PHC pharmacy. These costs were typically borne by the PHC HWs.

#### Unequal access to information within the community.

Participants reported concerns regarding access to information about the intervention among beneficiaries because of language barriers^§^ and the complex social structure in the rural communities, in which men had more exposure to messages than women. Understanding intervention details, including the timing, duration, and target age groups, varied according to community members’ age, gender, and social position. Interviews revealed that men, especially those between 25 and 35 years of age, were more aware than women of the conditions under which the intervention was designed, set up, and launched. In contrast, a large proportion of women in this age group were unaware that this was a trial. These inequalities stemmed from how information circulates locally and how important decisions are made in the village (i.e., mostly by men during meetings called kabarin-dehilahy [meetings between men]).

## DISCUSSION

Madagascar is looking to integrate novel strategies into its National Malaria Strategic Plan (NMSP) to reverse the increase in malaria incidence and mortality in recent years. In particular, age-expanded mCCM was included in the 2018–2022 NMSP to increase access to prompt malaria diagnosis and treatment among remote populations. To assess the efficacy, acceptability, feasibility, and cost-effectiveness of this strategy, a cluster randomized trial was done before a scale-up. As the Ministry of Public Health progressively scales up the age-expanded mCCM across Madagascar, this information regarding administrative and community realities will be critical to their success.

The results of qualitative data showed that mCCM was acceptable to most of those who were directly affected, including beneficiaries, HWs, and CHWs. High acceptability reflected that providing malaria care to older children was already an informal CHW practice that addressed critical community needs; formalizing the practice reduced the uncomfortable tensions between CHWs and the communities that they served related to restrictive policies. Although these results suggest that a scale-up is desirable, challenges identified in the study provide a road map for adapting the intervention during a scale-up.

Geographic proximity and ease of payment have been reported as key determinants of CHW utilization elsewhere in Madagascar,^28^^–^^30^ and other studies have identified social proximity as a major determinant of health care-seeking behavior.^31^ Here, social proximity between CHWs and community members contrasted with PHC staff members, who were widely viewed as outsiders, led to a general preference for CHWs.

Community members expressed confidence that CHWs were well trained and able to competently provide care for uncomplicated malaria to all community members. Furthermore, the age limitation imposed by the iCCM program led to pressure on CHWs to provide unauthorized care for older children and adults^28^; in the intervention arm, this problem was resolved and could explain the immediate and sustained tripling of CHW consultations observed.^8^

This study identified several threats to a successful age-expanded mCCM program. For example, CHWs need reliable and adequate supplies to test and treat all ages to induce and sustain a change in care-seeking practices. The age-expanded mCCM trial consistently reduced stock outs of malaria commodities at the community level, but these did occasionally occur and will likely be more common outside of a controlled study setting. Supply management is an ongoing challenge in Madagascar, placing CHWs in difficult situations in which they have to modify practice guidelines, adjust established practices, and pass the costs of supplies bought elsewhere to patients.^32^ Charging for supplies that are meant to be distributed free of charge has been observed elsewhere,^33^ and the costs passed to patients can be a barrier to care seeking.^28^^,^^34^ During the Farafangana trial, CHWs who charged for what should have been free commodities justified this practice because the excessive workload prevented them from pursuing income-generating activities and because they felt that some patients had the means to pay for services or supplies. In Madagascar, CHWs do not receive a salary for their service. They reported feeling torn between their commitment to malaria management, concern for the sick, fear of reprisals and revenge from community members if there were a death because of refusal of care,^¶^ and a lack of financial incentives. Some CHWs in the intervention arm hired people to work their land, and others complained of not even having time to cook their meals. Despite this, CHWs declared that they were generally satisfied with the intervention and would like to continue managing malaria in older children and adults. Thus, finding ways to financially compensate CHWs for their work, in line with WHO recommendations^1^ is necessary so that costs are not passed on to local populations in need of malaria care.

Malaria perceptions, knowledge, and practices observed here were consistent with those previously reported in Madagascar. The lack of knowledge about malaria and the nonspecificity of symptoms can lead to confusion with other diseases, such as flu,^35^ and malaria can be associated with witchcraft.^28^^,^^35^ Before the age-expanded mCCM trial, rural communities in Farafangana followed practices regarding malaria care seeking in line with these perceptions, such as self-medication, consultation with ombiasy (traditional healers), and the use of medicinal plants.^28^^,^^35^ These data demonstrated an evolution in malaria perceptions from being “socially nonexistent” in individuals older than 5 years old to its “social birth.” The social birth of an illness is the creation of new perceptions of an illness that was already present in the community.^18^ The qualitative data revealed how the iCCM program led to malaria being seen as a “childhood disease,” whereas the age-expanded mCCM raised it to the rank of a “clinical disease” affecting all age groups. As a result, ombiasy were perceived to have lost their power to cure malaria in the eyes of community members and were supplanted by CHWs, who were considered better equipped to detect and cure the disease. As a result, therapeutic meandering^35^ was reduced after the introduction of the intervention according to many accounts.

Although the primary objective of this research was to assess the acceptability of expanding mCCM to all ages, our findings, derived from an inductive approach, have sparked reflections on the methodologies used in clinical trials, especially RCTs. As a reminder, the management of malaria by CHWs for CU5 was implemented well before, and the aim of the mCCM trial was to test the expansion to those older than 5 years old, including adolescents and adults. The mCCM study seems to have enhanced tensions among community members who were divided into two groups during 14 months of implementation. The communities designated as the “intervention arm” are perceived as privileged by local actors, whereas those in the “control group” remained in their initial status. It is essential to highlight that the distinction between the Ministry of Health and the Institut Pasteur de Madagascar was not clear to community members; there was a misunderstanding of the experimental nature of the mCCM study, and the motivations behind the differentiation of communes were opaque to them or became increasingly unclear over time. The extension of community management to those older than 5 years old is a continuation of pre-existing practices, but the intervention, by dividing the communities into two groups, formalized this practice in one while marginalizing it in the other, leading to tensions. This phenomenon during RCTs is not isolated as similar cases have been reported.^36^ Ethical questions are generally raised by authors who suggest considering alternative methodologies^37^ or better communicating the contours of the intervention while emphasizing the experimental nature of the approach.^36^ In the case of the mCCM intervention, adopting the RCT methodology was deemed important to quantify differences (resources, care seeking, rates of diagnosis and treatment, malaria prevalence, etc.) in the effects of the initial policy and those of the age expansion. Information about the start and end of the intervention as well as the experimental dimension was communicated at the beginning of the intervention but could have faded from the beneficiaries’ memory over time. We, therefore, suggest that in this type of district-wide clustered intervention, this should be minimized through constant reminders and through ensuring that CHWs and community members are well aware of the experimental dimension. Interestingly, this tension and the constant demand from community members and CHWs in the control group to benefit from the intervention are good indicators of the acceptability of the age expansion itself.

Our study had several limitations. First, participant selection was not random. Although this is common in qualitative studies, it can lead to anchoring bias.^18^ This was minimized by choosing fokontany leaders rather than CHWs as community liaisons because part of the investigation was on the practices of CHWs. Second, research teams from Institut Pasteur de Madagascar were involved in intervention implementation, and they supported many activities, such as CHW supervision, supply chain strengthening, and reporting. Although qualitative study teams were different from the trial implementation and supervision teams, they worked for the same institution and could have biased participant responses. To limit this risk, an immersion approach^‖^ was used to establish a climate of trust. Third, data collection was conducted near the end of the intervention, which could have led to recall bias, particularly regarding events from the early days of the intervention. However, this timing allowed for the incorporation of a more comprehensive set of questions and concerns, enabling the qualitative team to consider all aspects of the intervention. This resulted in a more nuanced understanding of the acceptability and feasibility of age-expanded mCCM, which is necessary to guide nationwide scale-up. Finally, results concerning the positive effects of the intervention must be interpreted within the precise context of this study, which included a strong on-site presence of the research teams to support implementation.

## CONCLUSION

In conclusion, age-expanded mCCM was widely accepted by rural communities, CHWs, and HWs in Farafangana District. Accessibility to care for older children and adults resolved tensions between CHWs and the communities that they served and reduced pressure on CHWs to practice outside of their scope. Acceptability was also linked to a decrease in therapeutic meandering and self-medication. Age-expanded mCCM improved the knowledge of malaria in the community and contributed to an evolution of malaria perceptions from a childhood disease to a disease that affects all individuals. The main challenges that will need to be addressed during a scale-up include assuring continuous availability of malaria commodities at the community level, frustration of beneficiaries at being charged for malaria care that should be free, and the financial burden of age-expanded mCCM on CHWs and HWs.

## Supplemental Materials

10.4269/ajtmh.24-0678 Supplemental Materials

## Footnotes

^†^
“Mamo” is an adjective used to describe a drunk person. Equating the term with malaria refers to the wobbly gait of malaria sufferers, who sometimes feel dizzy.

^‡^
“Fanintona” is a word used to describe all kinds of epileptiform seizures.

^§^
Even if the research team is of Malagasy nationality but from a different region, the difference in dialect exists and still constituted a barrier.

^¶^
Our interview with one HW recounted the case of a CHW about 10 years ago whose house was burned down by people out of revenge. Although this was an isolated event, CHWs seem to fear that it could happen again.

^‖^
We stayed on site for several days so that the community could get used to our presence and trust us.
