# Supplementary material for: An Anthropological Analysis of Acceptability and Feasibility of Expanding Community-Based Malaria Management to All Ages in Madagascar: Levers and Challenges for National Scale-Up
Source: Am J Trop Med Hyg. 2025 Aug 12;114(2 Suppl):4–13. doi: 10.4269/ajtmh.24-0678 (PMC12874809; doi:10.4269/ajtmh.24-0678)
Supplement: Supplemental Materials [file tpmd240678.SD1.pdf]

Annex 1: Interview Guide with Community Health Workers in the Control Arm

(The major themes are written in bold, while the numbered questions were generally the follow-up questions if not addressed upfront in the answer)

Identification of the CHW
1- Age, participant identifier
2- Name of the fokontany where you are a CHW ?

History of their careers as CHW
3- How long have you been a CHW? How were you elected?
4- Do you remember the first project you worked on? If so, which one was it? And what did you do as a CHW in that intervention?

Training of the CHW
5- Did you receive training to be a CHW?
6- For the malaria project, were there any trainings? If yes, how often? What were the contents?
7- Who facilitated the training?
8- How do you evaluate the content of the training?
9- Do you have any additional training requests, especially regarding malaria?

Daily life as CHW
10- Can you describe your typical day? Your week ?
11- Can you share with us a little anecdote or story that has marked you as a CHW
12- What are your other functions (remunerated) in life?
13- In terms of availability, how many hours per day or per week do you dedicate to your role as a CHW?

Relationships with Facilitators for Malaria
14- Who are the NGOs, associations, etc., that are active for malaria in your fokontany?
15- How would you evaluate the cooperation and relationship between CHWs and these NGOs? Do they help you? What kind of support do they provide?

Other projects the CHW is responsible for
16- What are your other responsibilities in other projects as a CHW?
17- If there are several projects: Which project are you most comfortable with? Which one seems the easiest to you, and why?
18- If we compare quickly, what do the other projects bring more or less compared to this intervention?

Motivations of the CHW and their perception of their work and role
19- In your opinion, what is your role as a CHW?
20- How do you think people perceive your work as a CHW?
21- What motivates you as a CHW? (remuneration? social status? something else?)
22- What demotivates you as a CHW?

How the CHW perceives malaria as a disease
23- Is malaria a common disease in the region?

24- What do you know about the effects of malaria if it is not treated for children under 5 years, children over 5 years, and adults?
25- How would you define your role in managing malaria? (diagnosis, treatment, counseling)
26- Can you recount a notable or problematic event related to malaria that has impacted you?

Good practices in handling RDTs
27- When a patient comes to see you, can you describe how the management process goes from start to finish? What are the most important steps? Why is this important?
28- How do you perceive handling the RDT? Easy? Difficult? (if difficult, what aspects challenge you?), Have you ever encountered specific problems while handling it?
29- Do you check the expiration date of the kit each time before using it? What happens if it is expired when you open it?
30- Under what circumstances do you need to read the instructions? Does it pose any particular problems for you? (wasting time, difficulty in understanding…)
31- What would you like to improve in the kit we are currently using?

People's behavior towards Malaria
32- How many feverish children/adults do you see in a normal week? And how many have positive tests?
33- Generally, how long after the onset of symptoms do patients come to see you?
34- According to your experience, what difference, if any, is there between children over 5 years old and adults in terms of seeking help for fever among people in Farafangana regarding sensitivity, transmission, and prevention? (If there is a difference: medicinal plants, self-medication...) If self-medication is mentioned, where do they buy it?
35- Are there any taboos related to malaria in the region? If so, what are the impacts on your work or on the fight against malaria in general?

Awareness campaign
36- What kind of awareness messages about malaria are conveyed here in Farafangana? What are the contents of these awareness messages? How do people respond to them?

Relationship with newcomers to the population
37- How do the people of Farafangana react to individuals who are not from the region? (CSB chief, foreigners like IPM missionaries, etc.)

Challenges in malaria management
38- What problems do you encounter in managing malaria?
39- What behaviors of people make it difficult for CHWs to provide care?
40- In your opinion, what factors encourage/discourage community members from seeking care from CHWs?

Relationship with other institutions:
41- How often do you receive a supervisory visit? How does this type of visit go? What is done during this kind of supervision? Who conducts it, especially regarding malaria?
42- How would you describe your relationship with the nearest CSB? (Do you have the necessary support? Have you had any particular issues?) → [remind of total confidentiality of the study if hesitation is sensed]

43- Are there certain types of support from the health structure that you would like to receive but are not currently getting? What types of support?
44- In your opinion, do people tend to go to CHWs or to CSBs? Why?

Reporting (case registration)
45- Reporting: how often do you do it? Do you do it every time or only under certain circumstances?
46- What do you think is the importance of reporting cases at the CSB level?
47- Are there any particular difficulties regarding the reporting of cases?

Capacity for expansion
48- Do you think that older children (> 5 years) and adults, if allowed, would want to come to you for treatment if they have a fever? Why? Why not?
49- Do you have the capacity to care for older children and adults with malaria? What would you need to succeed

Do you have anything else to add ?

Annex 2: Interview Guide with Community Health Workers in the Intervention Arm

(The major themes are written in bold, while the numbered questions were generally the follow-up questions if not addressed upfront in the answer)

Identification of the CHW
1- Age, participant identifier
2- Name of the fokontany where you are a CHW ?

History of their careers as CHW
3- How long have you been a CHW? How were you elected?
4- Do you remember the first project you worked on? If yes, which one? What was your role as a CHW in that intervention?

Training of the CHW
5- Did you receive training to become a CHW?
6- For the malaria project, were there any trainings? If yes, how often? What were the contents?
7- Who facilitated the training?
8- How do you evaluate the content of the training?
9- Do you have any additional training requests, especially regarding malaria?
10- What demotivates you as a CHW?

How the CHW perceives malaria as a disease
11- Is malaria a common disease in the region?
12- What do you know about the effects of untreated malaria on children under 5 years, children over 5 years, and adults?
13- How would you define your role in managing malaria? (diagnosis, treatment, counseling)
14- Can you recount a notable or problematic event related to malaria that impacted you?

Daily life as CHW
15- Can you describe your typical day? Your week ?
16- Can you share with us a little anecdote or story that has marked you as a CHW ?
17- What are your other functions in life? (remunerative)
18- In terms of availability, how many hours per day or per week do you dedicate to your role as a CHW?

Evaluation of the Intervention
19- What does the new intervention for malaria management in your municipality consist of?
20- How was this intervention concretely implemented? Were you supported? Did you receive additional training in this regard?
21- What is your opinion on this intervention? Were the objectives clear? If not, how would you clarify them?
22- How have people responded to this intervention? What do they generally think about it?
23- Has the existence of this change affected your daily life, attendance, stock management, etc.? Has it generated any difficulties?
24- Have you encountered difficulties in managing those over 5 years? Would you like to continue with this management?
25- Do you have any suggestions for improvement? Do you have anything to say?

Other projects the CHW is involved in

26- What are your other responsibilities in other projects as a CHW?

27- If there are several projects: Which project are you most comfortable with? Which seems the easiest, and why?

28- If we make a quick comparison, what do the other projects offer more or less compared to the malaria project?

Relationships with Facilitators for Malaria

29- Who are the NGOs, associations, etc., that are active for malaria in your fokontany?

30- How would you evaluate the cooperation and relationship between CHWs and these NGOs? Do they help you? What kind of support do they provide?

Motivations of the CHW and their perception of their work and role

31- In your opinion, what is your role as a CHW?

32- How do you think people perceive your work as a CHW?

33- What motivates you as a CHW? (remuneration? social status? something else?)

Good practices for handling the RDT

34. When a patient comes to see you, can you tell us how the management process goes from start to finish? What do you think are the most important actions? Why is this important?

35. How do you perceive the handling of the RDT? Easy? Difficult? (If difficult, what aspects do you find challenging?) Have you ever encountered any particular problems while handling it?

36. Do you check the kit's expiration date every time before using it? What happens if it is expired when you open it?

37. Under what circumstances do you need to read them? Does it pose any particular problems for you? (Time-consuming, difficulty in understanding…)

38. What would you like to improve in the kit we are currently using?

People's behavior towards malaria

39. How many children/adults with fever do you see in a normal week? And how many have positive tests?

40. Generally, how long after the onset of symptoms do patients come to see you?

41. Based on your experience, what differences, if any, are there between children over 5 years old and adults in terms of seeking care for fever in people in Farafangana regarding sensitivity, transmission, and prevention? (If there is a difference: medicinal plants, self-medication…) If self-medication is mentioned, where do they buy it?

42. Are there any taboos related to malaria in the region? If so, what are their impacts on your work or on the fight against malaria in general?

Awareness campaign

43. What kind of awareness messages about malaria are made here in Farafangana? What are the contents of these awareness messages? How do people respond to them?

Relationship with Newcomers to the Population

44. How do the people of Farafangana generally react to individuals who are not from the region? (CSB chief, foreigners such as IPM missionaries, etc.) (Skeptical? Welcoming?)

45. Have they been enthusiastic about the changes in the extension of malaria management?

Problems in Managing Malaria

46. What problems do you encounter in managing malaria? (Lack of supplies, reluctance of the population to adhere to treatments, delays in seeking care, self-medication…)
47. What behaviors of people make it difficult for CHWs to provide care?
48. In your opinion, what factors encourage/discourage community members from seeking care from CHWs?

Relationship with Other Institutions:

49. How often do you receive a supervisory visit? How does this type of visit go? What happens during this kind of supervision? Who conducts it, especially regarding malaria?
50. How would you describe your relationship with the nearest CSB? (Do you have the necessary support? Have you had any particular issues?) → [Remind about the total confidentiality of the study if there is hesitation]
51. Are there certain types of support from the health structure that you would like to have but are not currently receiving? What types of support?
52. In your opinion, do people tend to go to CHWs or to CSBs? Why this preference?

Reporting of Cases

53. Reporting: how often do you do it? Do you do it every time or only under specific circumstances?
54. What do you think is the importance of reporting cases at the CSB level?
55. Are there any particular difficulties regarding the reporting of cases?

Do you have anything else to add ?

Annex 3: Interview Guide with Health Personnel in Control Arm

(The major themes are written in bold, while the numbered questions were generally the follow-up questions if not addressed upfront in the answer)

Participant Identification
1. Health facility affiliation
2. Age, participant identifier
3. Type of provider (doctor, nurse, health agent, etc.)
4. How long have you been working here? Where did you work before? Can you describe your typical day ?
5. When did you last receive training on malaria case management? Who (organization or entity) provided the training?
6. Can you share with us a little anecdote or story that has marked you in your role ?

Malaria
7. Is malaria a common disease in the region?
8. How would you define your role in malaria management? (diagnosis, treatment, counseling)
9. Can you recount a notable or problematic event related to malaria that impacted you?

People's Behavior Towards Malaria
10. Based on your experience, what is the trend of healthcare-seeking behavior among the people of Farafangana in cases of fever in young children (under 5 years, over 5 years)? (self-medication, medicinal plants, medications: where do they obtain them?)
11. Based on your experience, is there a difference, if any, in the response to fever between children over 5 years and adults among the people in Farafangana in terms of sensitivity, transmission, and prevention? (if there is a difference: medicinal plants, self-medication…) If self-medication is mentioned, where do they buy them?
12. Are there any taboos related to malaria in the region? If so, what are the impacts on your work or on malaria control in general?
13. Are there practices and taboos that make it difficult to combat malaria?

Interaction with CHWs
14. In general, what is the role of CHWs? Especially regarding malaria?
15. How would you describe the relationship you have with CHWs? Do you know one or two in particular?
16. Regarding malaria, what is the difference between management by CHWs and at the CSB level? Are there specific cases that cannot be managed by CHWs? How does this generally work?
17. How would you evaluate the management by CHWs for children under 5 years?
18. Can you provide some examples to illustrate your relationship (stories of collaboration in caring for a sick patient)?
19. Do you have a responsibility towards CHWs? (training, follow-up, supervision, support). If yes, please describe these responsibilities.
20. (For those providing supervisory support) How often do you provide supervisory support to CHWs?
21. (For those providing supervisory support) What is the supervision process? What is examined during supervision? How often do CHWs submit records? As often as required? If not, why do you think that is, where lies the difficulty?

22. Do you think people trust their CHWs? If yes, why? If no, why not?
23. How are CHWs useful to your work? Do CHWs lighten your workload? If yes, how?
24. Do CHWs add to your workload? If yes, how?
25. Have the CHWs you work with managed to complete all the tasks they were supposed to do?

Awareness Raising
26. Who conducts awareness-raising on malaria in Farafangana? (NGOs? associations?), in what format are these awareness sessions? What are the roles of these entities in the fight against malaria? How do they work with you?
27. What messages are contained in these awareness sessions? How do people perceive them, in your opinion?

Attitude Towards Newcomers in the Population
28. How do the people of Farafangana generally react to individuals who are not from the region? (CSB chief, foreigners like IPM missionaries, etc.) (suspicious? welcoming?)
29. How did community members in this commune take the news of changes due to intervention in other communes? How do they feel about this difference

Management of Malaria Cases
30. In your opinion, what problems do CHWs encounter in malaria management?
31. What problems do you encounter in managing malaria? (lack of supplies, reluctance of the population to follow treatments, non-compliance with treatment duration, delays in seeking care, self-medication)
32. Generally, how long after the onset of symptoms should patients come to see you in case of malaria?
33. In your opinion, what factors encourage community members to seek treatment for fever?
34. In your opinion, what factors discourage community members from seeking care for fever?
35. What could be / what were the advantages of managing malaria cases at all ages by CHWs at the community level?

Capacity for expansion
36. What is your overall opinion on the intervention allowing the treatment of older children and adults with fever at the community level?
37. Do you think that CHWs are sufficiently competent and trained for this extension of management? If yes/no, why?
38. Do you have any suggestions for improvement or anything to add?

Annex 4: Interview Guide with Health Worker in the Intervention Arm

(The major themes are written in bold, while the numbered questions were generally the follow-up questions if not addressed upfront in the answer)

Participant identification
1. Health facility affiliation
2. Age, participant identity
3. Type of provider (doctor, nurse, health worker, etc.)
4. How long have you been working here? Where did you work before? Can you describe your typical day ?
5. When did you last receive training or updates on malaria case management? Who (organization or entity) provided the training?
6. Can you share with us a little anecdote or story that has marked you in your role ?

Malaria
7. Is malaria a common disease in the region?
8. How would you define your role in malaria management? (diagnosis, treatment, counseling)
9. Can you recount a notable or problematic event related to malaria that has impacted you?

People's behavior towards malaria
10. Based on your experience, what is the trend in how people in Farafangana seek care for fever in young children (under 5 years, over 5 years)? (self-medication, medicinal plants, medications: where do they obtain them?)
11. Based on your experience, is there a difference, if any, in the response to fever between children over 5 years and adults in Farafangana in terms of sensitivity, transmission, and prevention? (if there is a difference: medicinal plants, self-medication…) If self-medication is mentioned, where do they purchase them?
12. Are there any cultural taboos related to malaria in the region? If so, what are the impacts on your work or on malaria control in general?
13. Are there practices and taboos that make it difficult to combat malaria?

Awareness Raising
14. Who conducts malaria awareness campaigns in Farafangana? (NGOs? associations?) What roles do these entities play in the fight against malaria? How do they work with you?
15. What messages are included in these awareness campaigns? How do people perceive them, in your opinion?

Interaction with CHWs
16. In general, what is the role of CHWs? Especially regarding malaria?
17. How would you describe your relationship with CHWs? Do you know one or two in particular?
18. Regarding malaria, what is the difference between care provided by CHWs and at the CSB level? (Are there specific cases that cannot be managed by CHWs? How does this generally work?)
19. How would you evaluate the care provided by CHWs for children under 5 years old?
20. In your opinion, would an extension of care to those over 5 years old, including adults, be feasible for CHWs? If yes or no, please explain why?
21. In your view, what problems do CHWs encounter in managing malaria?

22. Can you provide some examples to illustrate your relationship (stories of collaboration in caring for a sick patient)?
23. Do you have any responsibilities towards CHWs? (training, monitoring, supervision, support). If yes, please describe these responsibilities.
24. (For those providing supervisory support) How often do you provide supervisory support to CHWs?
25. (For those providing supervisory support) What is the supervision process? How often do CHWs submit records? As often as required? If not, why do you think that is, where do you see the difficulty?
26. Do you think people trust their CHWs? If yes, why? If no, why?
27. How are the CHWs useful to your work? Do the CHWs lighten your workload? If so, how?
28. Do the CHWs add to your workload? If so, how?
29. Have the CHWs you work with managed to complete all the tasks they were supposed to do?

Report to newcomers in the population
30. In your opinion, how do the people of Farafangana generally react to individuals who are not from the region? (CSB leaders, foreigners such as IPM missionaries, etc.) (suspicious? welcoming?)

Management of malaria cases
31. What problems do you encounter in managing malaria?
32. Generally, how long after the appearance of symptoms do patients need to come see you in case of malaria?
33. In your opinion, what factors encourage community members to seek treatment for fever?
34. In your opinion, what factors discourage community members from seeking care for fever?

Understanding and evaluation of the intervention
35. What does the new intervention for malaria management in your municipality consist of? (management for those over 5 years old). Since when has this change been effective?
36. What is your opinion on this intervention?
37. How have people responded to this intervention? What do they generally think about it?
38. Has the existence of this change affected your daily life, patient influx, stock management, etc.?
39. Do you think that the intervention which expanded malaria management in the community to all ages will increase the number of diagnosed and treated malaria cases?
40. Do you have any suggestions for improvement regarding this extension of care?
41. Do you think that the extension of community management of malaria cases to all ages will improve the diagnosis and treatment of malaria? • If yes, why? • If no, why?
42. What could be / what were the benefits of managing malaria cases at all ages by CHWs at the community level?
43. What has been the impact of this intervention on your daily life / workload? Can you describe these impacts?
44. What is your overall opinion on the intervention allowing the treatment of older children and adults with fever at the community level?
45. Do you have any suggestions for improvement or anything to add?

Annex 5 : Interview Guide with Community Members

A series of additional questions has been added for community members in the Intervention Arm when the interviewee is able to formally identify in which arm their community is located and perceives the differences between the two arms

Name :
Age :
Gender :
Village name :
Main activity :
Number of children :
Age of children :
Marital status :
Distance from the residence to the nearest health center (in hours or walking duration) :

Introduction to the Disease

1. Can you list the most common diseases in your area?
2. Which ones are the most serious? Why? In what sense?

Behavior in Case of Fever

<u>Scenario A): If the person has one or more children under 5 years old</u>

3. What do you do for your child if he or she has a fever? Who do you see first?
4. How long do you generally wait before taking this action?
5. What justifies this action for you? Who makes the decision within the household?
6. Up to what age do you plan to apply this principle?
7. For yourself, when you have a fever, what are your first actions and initial recourse? Who do you go to? Why?
8. Do you have a preference between going to the CHW or going to the CSB? Why?

<u>Scenario B): If the person has children of different age groups: over 5 years old and under 5 years old</u>

9. Is there a difference in your approach to care or the treatments you give to your two children when they have a fever?
10. What are the treatments for the older children, and for the younger ones? Why is there this difference, if any?
11. Do you have a preference between going to the CHW or going to the CSB? Why?

<u>Scenario C): If the person is a parent of a child aged 5-15 years</u>

12. When your child was under 5 years old, what was your first recourse in case of fever?
13. Now that he/she is … years old, have you changed your method when he/she has a fever? If yes, what justifies this change for you?
14. What methods are used by these different actors (proposed treatments)? What type of care/treatment do they offer?
15. Do you have a preference between going to the CHW or going to the CSB? Why?

Perceptions and Representations of “Fever” and “Malaria”

16. What is malaria? (Please specify the terminology for reference: if we talk about "tazo"/"tazomoka"/or another name and what the term corresponds to).
17. When did malaria first appear in your village?
18. Can you describe the symptoms of malaria ?
19. What are its causes? (The concept of contagion? Link with the mosquito?)
20. If the mosquito is mentioned as a cause: is it possible to differentiate between a fever caused by a mosquito and other types of fevers? If yes, how? If no, why? Distinguish between diagnosed malaria based on clinical symptoms and a diagnosis confirmed by a test (RDT).

Treatment (in case of fever related or not to malaria)

21. Are there treatments for malaria? What do we call these treatments? (infusions, paludar, palustop, ACT)?
   - Where can we obtain them (grocery store, market, from the CHW, health agent)?
   - At what price?
   - Are they available without a prescription?
22. Who advised you to take these medications? If they were prescribed, who was the prescriber?
23. If the person takes ACT: how is it? In what cases do you take it? Where can I obtain it? At what price? If prescribed, who is the prescriber?

Acceptance and understanding of the Intervention (For Intervention Arm, or adapt to Control arm on the field if necessary)**

24. What does the new intervention for malaria management in your community consist of? (Management for those over 5 years old). Do you feel concerned as an adult?
25. How did you learn about this change? (Mass awareness, home visits, presentation by the study/intervention team)
26. What is your opinion on this extension of care for those over 5 years old?
27. As an adult, have you benefited from this change in management? How did it go? What did you think about it? If yes, would you continue? If no, why?
28. How has this intervention impacted your life and where do you seek care for yourself and your family? Can you describe these impacts?
29. Before this intervention, what did you do? Can you give us an example?
30. Overall, what is your opinion on the intervention involving CHWs taking care of older children and febrile adults? Any suggestions for improvement?
31. Do you think that case management of malaria in all communities, regardless of age, would be effective in all communities?
    - If no, why not?
    - If yes, why?
    - Are there communities where this might work better?

Perception of the role of CHWs

31. If CHWs were not mentioned during the interview: what is the role of the CHW in your fokontany? Who are they? Do you know them well? Have you met them often? Does the CHW have any particular authority within the community? If so, what type of authority? Can you give examples?
32. What kind of services do CHWs provide to your community? Have you ever sought help from CHWs for any of these services? Why/why not?

33. If CHWs were not mentioned earlier in the management of malaria: what do they do to help you in the event of a malaria episode? (Describe the activities in detail and emphasize understanding of the RDT, ACT, referral to a health center, etc.)
34. What was the procedure for managing a case of malaria by a CHW? What was the procedure for getting tested for malaria? What was the process for receiving a diagnosis for you (or your family member)? What was the procedure for receiving treatment for malaria?
35. Are they available when you need them? Do they provide you with information about your health condition, the test, or the treatment? Are you satisfied with their work?
36. What facilitated your choice to seek care from CHWs in the case of malaria? What prevented you from using CHWs for malaria control services?
37. Are there any additional services you would like CHWs to provide?
38. How would you describe your relationship with the CHW (trust, authority)?
39. Do you have anything else to add ?

Annex 6 : Interview guide with mCCM-supervisors

1) What Commune are you in charge of?
2) What can be the particularity of this Commune compared to others ?
3) Can you describe your responsibility in this project?
4) What is your typical day as a supervisor?
5) In your opinion, what is the relationship of the communities here with malaria?
6) How would you describe your work? (follow-ups: difficulties, satisfactions, etc.)
7) How would you describe the competence of the CHWs?
8) How would you describe your relationship with the CHWs? the HWs? the local communities?
9) How would you describe the relationships between the CHWs and the HWs? And between the CHWs and the communities? And between the HWs and the communities?
10) What do you think of the mCCM project? (in relation to malaria, relationships, usefulness, etc.)
11) Based on your experiences in this project or elsewhere, what is your recommendation for an intervention of this type?
12) What do you think about the acceptability of this intervention among the beneficiaries?
13) Can you share a small anecdote or story related to malaria that has impacted you or that you experienced while working on this intervention?
14) What can you tell us about the supply chains? (follow-up: difficulties, improvements)
15) What difference do you perceive between the Control arm and the Intervention arm? Can you explain?
16) How would you evaluate the communication in this intervention? Positive points? Points to improve?
17) How would you evaluate the understanding of the communities and the CHWs regarding this intervention?
18) Do you have anything else to add?

Annex 7 : Focus Group with Community Health Member in the Interventionl Arm

1) **What can you say about malaria ?**

⇨ Follow-up

What do you know about malaria?
In your opinion, what do community members know about malaria? How do they perceive this disease based on your observations?
Which age group do you think is the most vulnerable to malaria according to your observations? What do you base this conclusion on?
What are the challenges in managing malaria?

2) **How is your (daily) life as CHW : can you describe beeing an CHW ?**

⇨ Follow-up

How long have you been a CHW in this village?
How is it going? What are your challenges?
(Try to delve into the difficulties encountered before and after mCCM or those that have not been resolved by mCCM.)

3) **How do you define your role in managing malaria ?**

⇨ Follow-up

In the mCCM project and outside of the mCCM project.
Before mCCM and after mCCM.

4) **Can you please describe your relationship with the Health Center (CSB) ?**

⇨ Follow-up

At the time of the survey.
Before mCCM.
Since mCCM.
What works well, and what needs improvement? Why?
Any anecdotes or stories if possible.

5) **Can you talk about your relationship with Community Members ?**

⇨ Follow-up

What is your relationship with community members?
How would you evaluate your relationship with community members?
Has this relationship with the community evolved recently, or has it remained the same? If there has been an evolution, can you pinpoint when?

Since when have they concretely taken care of children over 5 years old, adolescents, and adults?

⇨ Follow-up

Can you tell us about the first time you took care of a person over 5 years old? How did it go? What were your feelings?

6) **Can you describe your preparation for mCCM ? Can you describe how mCCM was first implemented in your region ?**

⇨ Follow-up

How did this preparation go?
Official and unofficial communication about the project.
The training they received.
Their understanding of the project.
Did they have doubts at the beginning? How have these doubts evolved?
Were they properly supported? By whom?

7) **Could you discuss how mCCM has impacted your life, work, and so on?**

⇨ Follow-up

Any stories and anecdotes on the subject?
Filling out registers.
Caring for patients.
Their work in the fields.
What are the positive aspects?
What aspects need improvement?

8) **What is your sentiment regarding the mCCM Project and the strategy for expanding care?**

⇨ Follow-up

When did this start?
What do you remember about its implementation?
Who approached you, and what were you told at that time?
How did you take it?
Have there been any changes since mCCM? If so, how are you experiencing them?
What are the positive points of the mCCM project according to your experience?
What are the negative points? Do you think these negative points are improvable? If yes, what are your suggestions for improvement?
The distribution of medicines and tests: have there been shortages? How many times? Can you recount? How was this resolved?

9) **In your opinion, is the strategy of expanding community care for malaria to all age groups, acceptable and feasible? Could you provide justification for yourresponse?**

⇨ Follow-up

How do you see the coming years as a CHW?
Do you think the expansion of care has been accepted? By you as a CHW? By the population? Why? What makes you say that?
Are there aspects to improve? To remove? To maintain? Explain.
Do you think you can continue providing care in the event that there is no compensation?

Do you think you can continue providing care if none of these improvements are made?
Do you have anything else to add ?

Annex 8 : Focus Group with Community Members in the Intervention Arm

1) Can you tell us about the IPM, its activities and your connection with this institution?

⇨ Follow-ups

Do you know about the IPM? (and what it does in your village?)
How long has the IPM been present in your village?

2) What can you tell us about malaria?

⇨ Follow-ups

What does malaria mean to you?
How long has malaria been present in your village?
If they say that malaria has appeared recently: What other disease that resembles malaria was present before malaria in the village? What are the main differences
between malaria and similar diseases?
What category of people do you think is vulnerable to malaria? Why?
What makes you sure that it is malaria affecting you or a family member?
What other diseases have (become or resemble) malaria?
What symptoms prompt you to seek medical help?

3) What is the evolution of malaria in your village?

⇨ Follow-ups

How long has malaria been present in your village?
What significant events concerning malaria in your village do you remember?

4) Can you share your personal experiences or observations concerning cases of malaria in your family or neighbourhood?

⇨ Follow-ups

How did it start? (symptoms)
During what period or date did this happen? (emphasize concrete events as reference points, e.g., elections, rainy season…)
What were the first actions or reflexes taken, and which person did the patient go to see?
What is the status of the person who took care of the patient (healthcare personnel? CHW? or others)? Why this choice?
What was the cost of the care?
How was the care provided? What was the composition of the treatment? Its duration?
How was the treatment followed up? Difficulties, etc.
How did it end?

5) **Can you tell us about the recourse and the treatment in case of fever in your community ?**

⇨ Follow-ups

Differentiate the recourse:
- For a child under 5 years old?
- For a child aged 6-14 years?
- For the adult themselves or another adult in their household?

6) **What can you share with us about the prevention of malaria ?**

⇨ Follow-ups

Do you think there are methods to prevent malaria? If so, which ones?
How did you learn about them?
Do you think they are effective?
How long have you been using them?

7) **What can you share with us about the treatment and care of malaria ?**

⇨ Follow-ups

What treatments cure malaria?
When does the community seek treatment?
Where do community members go for recourse in case of malaria?
What do you think about the malaria treatments provided by CHWs?
What do you think about malaria treatments provided elsewhere? (specifying where if the question arises)
What difficulties do you encounter in managing malaria? What solutions do you recommend?
What are the costs of treatment, and how do you perceive this cost?

8) **What can you tell us about self-medication for malaria?**

⇨ Follow-ups

Do you ever treat yourself on your own initiative? Why this choice? How did it turn out? What do you think of this method?
What is their opinion on self-treatment?
What do you think is the trend among community members regarding self-medication?

9) **Can you tell us about the mCCM intervention ?**

⇨ Follow-ups

What does mCCM mean to you?
What are your feelings about this system?
What has mCCM brought? (positives and negatives)
What do you think is the role of CHWs in mCCM? Have they succeeded?
How is your relationship with CHWs?
How is your relationship with the CSB staff?

10) **Can you describe the relationship between the community members and the CHWs in your village ?**

⇨ Follow-ups

Details about your CHW: who is your CHW? How is your relationship with this person?
How do you perceive the work of your CHW?
Has your relationship with the CHW evolved recently? If so, describe how and in what direction.
What do CHWs manage in your neighborhood? What do you think about that?
Who manages the CHWs in your neighborhood?
Since when has your CHW been responsible for children aged 6 years? Adolescents? Adults? Why these differences? (if applicable)
What do you think about this management by the CHWs?

11) Do you think this strategy mCCM was acceptable in your region ? Can you clarify your answer ?

⇨ Follow-ups

Do you accept the extension and continuity of the strategy for malaria management by CHWs to individuals over 5 years old? Why?
What do you think is good about what is already in place?
What needs improvement?

12) Do you have anything else to add?
